# A Case of Delayed-Onset Hyphema After PreserFlo MicroShunt Implantation

**DOI:** 10.7759/cureus.116822

**Published:** 2026-09-24

**Authors:** Yoshichika Wanami, Hinako Ohtani, Chisako Ida, Keigo Takagi, Yuto Yoshida, Kazunobu Sugihara, Masaki Tanito

**Affiliations:** 1 Department of Ophthalmology, Shimane University Faculty of Medicine, Izumo, JPN

**Keywords:** blood reflux, delayed-onset hyphema, glaucoma surgery, gonioscopy, preserflo microshunt, schlemm’s canal

## Abstract

We report a case of delayed-onset hyphema with reflux bleeding adjacent to the PreserFlo MicroShunt (PFM) tube entry site. A 66-year-old Japanese man with bilateral primary open-angle glaucoma underwent PFM implantation in the left eye because of elevated intraocular pressure (IOP) and progressive visual field loss despite treatment with four topical anti-glaucoma agents. He had previously undergone cataract surgery and microhook ab interno trabeculotomy (μLOT) in the same eye. Because of insufficient IOP control and further visual field progression after PFM implantation, Ahmed glaucoma valve implantation with pars plana tube insertion was subsequently performed in combination with 25-gauge pars plana vitrectomy. At two years and three months after PFM implantation, a small number of red blood cells were observed floating in the anterior chamber. Gonioscopy showed that the PFM tube was positioned between the pigmented and non-pigmented trabecular meshwork, with reflux bleeding from both sides of the tube entry site. The previous μLOT clefts did not overlap with the PFM tube entry site. The hyphema resolved with observation alone, although possible recurrence was noted six months later. This case demonstrates that delayed-onset hyphema can occur more than two years after PFM implantation and that careful gonioscopic examination can help identify the source of bleeding.

## Introduction

The PreserFlo MicroShunt (PFM; Santen Inc., Osaka, Japan) is a subconjunctival filtering device used for the surgical treatment of glaucoma. It lowers intraocular pressure (IOP) by draining aqueous humor from the anterior chamber into the subconjunctival space. A recent five-year study demonstrated sustained reductions in IOP and medication burden after PFM implantation, although some eyes required further glaucoma surgery [1]. In Japanese patients, a large multicenter study likewise demonstrated reductions in IOP and medication use at 12 months after PFM implantation [2]. During PFM implantation, anterior chamber entry is created at the level of the trabecular meshwork (TM), potentially placing the tube entry site near Schlemm’s canal (SC) [3]. This anatomical relationship is relevant when evaluating bleeding around the tube entry site. Proximity to or possible communication with SC may provide a pathway for blood to reflux into the anterior chamber through the tube entry site.

Compared with trabeculectomy, PFM implantation has been associated with a more favorable postoperative safety profile, although its IOP-lowering effect may be less pronounced [4]. Reported postoperative complications include hypotony, choroidal detachment, bleb-related complications, and tube obstruction. Additional interventions, such as needling or surgical revision, may be required in some cases [3,4]. Transient hyphema in the immediate postoperative period has also been reported [5]; however, delayed-onset hyphema associated with the PFM tube entry site has rarely been described. Delayed-onset hyphema has also been documented years after microhook ab interno trabeculotomy (μLOT), with blood reflux from SC proposed as a possible mechanism [6]. Unlike transient hyphema occurring in the immediate postoperative period, very late-onset hyphema presents the clinical challenge of determining which of the previously operated sites is the source of bleeding. Thus, in eyes with a history of multiple glaucoma procedures, careful examination is needed to distinguish potential bleeding sources at different surgical sites.

Herein, we report a case of delayed-onset hyphema occurring two years and three months after PFM implantation in an eye with previous μLOT and subsequent Ahmed glaucoma valve implantation. Gonioscopy revealed reflux bleeding adjacent to the PFM tube entry site, separate from the previous trabeculotomy clefts, with possible recurrence six months later. This case reports delayed-onset hyphema observed after PFM implantation and indicates that careful observation, particularly gonioscopic examination, may help identify the source of bleeding. The clinical relevance of this case lies in documenting a rare, very late episode of reflux bleeding localized by gonioscopy to the PFM tube entry site in an eye with a history of multiple glaucoma procedures.

## Case presentation

A 66-year-old Japanese man with bilateral primary open-angle glaucoma (POAG) was scheduled for PFM implantation in the left eye (OS) because of elevated IOP and progressive visual field loss despite topical medical therapy. He had undergone cataract surgery OS 10 years earlier and microhook ab interno trabeculotomy (μLOT) OS four years earlier. His systemic medical history included atrial fibrillation treated with catheter ablation. He was not taking anticoagulant medication. Before PFM implantation, best-corrected visual acuity (BCVA) OS was 1.2 (decimal notation) with a −1.50 D spherical correction. IOP OS, measured by Goldmann applanation tonometry (GAT), was 20 mmHg while receiving fixed-combination carteolol/latanoprost once daily and fixed-combination brinzolamide/brimonidine twice daily. The conjunctiva was quiet, the cornea was clear, and the intraocular lens was well positioned. The cup-to-disc ratio (vertical × horizontal) was 0.8 × 0.8. The mean deviation (MD) values on Humphrey 30-2 and 10-2 visual field testing (Humphrey Field Analyzer, Carl Zeiss Meditec, Dublin, CA, USA) were −9.18 dB and −16.86 dB, respectively.

During PFM implantation, a limbal conjunctival incision was made from the 9- to 12-o’clock positions. Sponges soaked in 0.04% mitomycin C were placed subconjunctivally for three minutes, followed by irrigation with 100 mL of balanced salt solution. A point 3 mm posterior to the limbus at the 11-o’clock position was marked. A scleral tunnel was then created smoothly on the first attempt from this point toward the presumed level of the pigmented TM using a double-step knife supplied in the PFM implantation kit. The pigmented TM was intentionally selected as the target of the anterior chamber entry site. The PFM was inserted through the tunnel without difficulty, and aqueous flow through the tube was confirmed. Tenon’s capsule and the conjunctiva were closed with 10-0 Vicryl sutures. Intraoperative hyphema, consisting of a small amount of blood reflux into the anterior chamber, was observed, and bleeding from the SC was suspected at that time, but no other intraoperative complications occurred. Postoperatively, 1.5% levofloxacin and 0.1% betamethasone phosphate eye drops were administered OS four times daily for one and three months, respectively. On the first postoperative day, only a few floating red blood cells and a small layered hyphema of less than 1 mm were observed in the anterior chamber. No postoperative elevation in IOP was observed. These findings resolved spontaneously within seven days after surgery. At 1.5 months after PFM implantation, IOP OS was 21 mmHg, and a needling procedure was performed. At one year and four months, IOP OS had increased to 29 mmHg, and a second needling procedure was performed. The MD on Humphrey 10-2 visual field testing had worsened to −24.26 dB. Therefore, Ahmed glaucoma valve (AGV) implantation in the superotemporal quadrant, with tube insertion through the pars plana, was performed in combination with 25-gauge pars plana vitrectomy one year and five months after PFM implantation. Three months after AGV implantation, BCVA OS was 0.8, and IOP OS was 9 mmHg while receiving fixed-combination brinzolamide/brimonidine twice daily.

**Figure 1:**
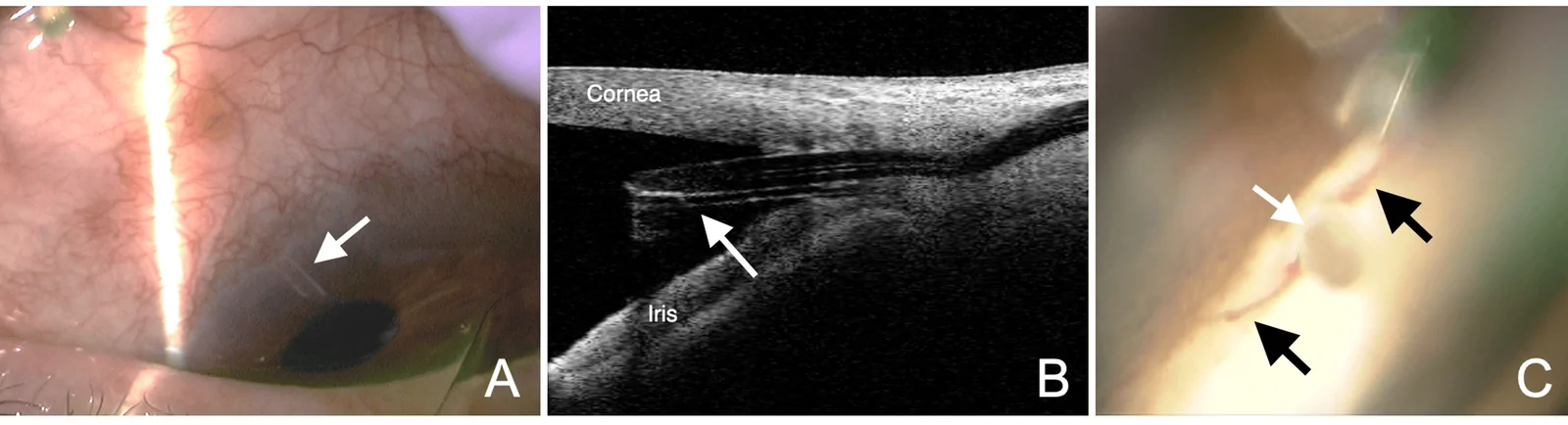
Findings in the left eye at two years and three months after PFM implantation. (A) Slit-lamp examination shows the PFM tube at the 11-o’clock position (white arrow) and a well-formed filtering bleb. (B) Anterior segment optical coherence tomography shows that the PFM tube (white arrow) appears largely unchanged in position compared with immediately after implantation. (C) Gonioscopy shows the PFM tube positioned between the pigmented and non-pigmented trabecular meshwork (white arrow), with reflux bleeding (black arrow) adjacent to the tube entry site.

**Figure 2:**
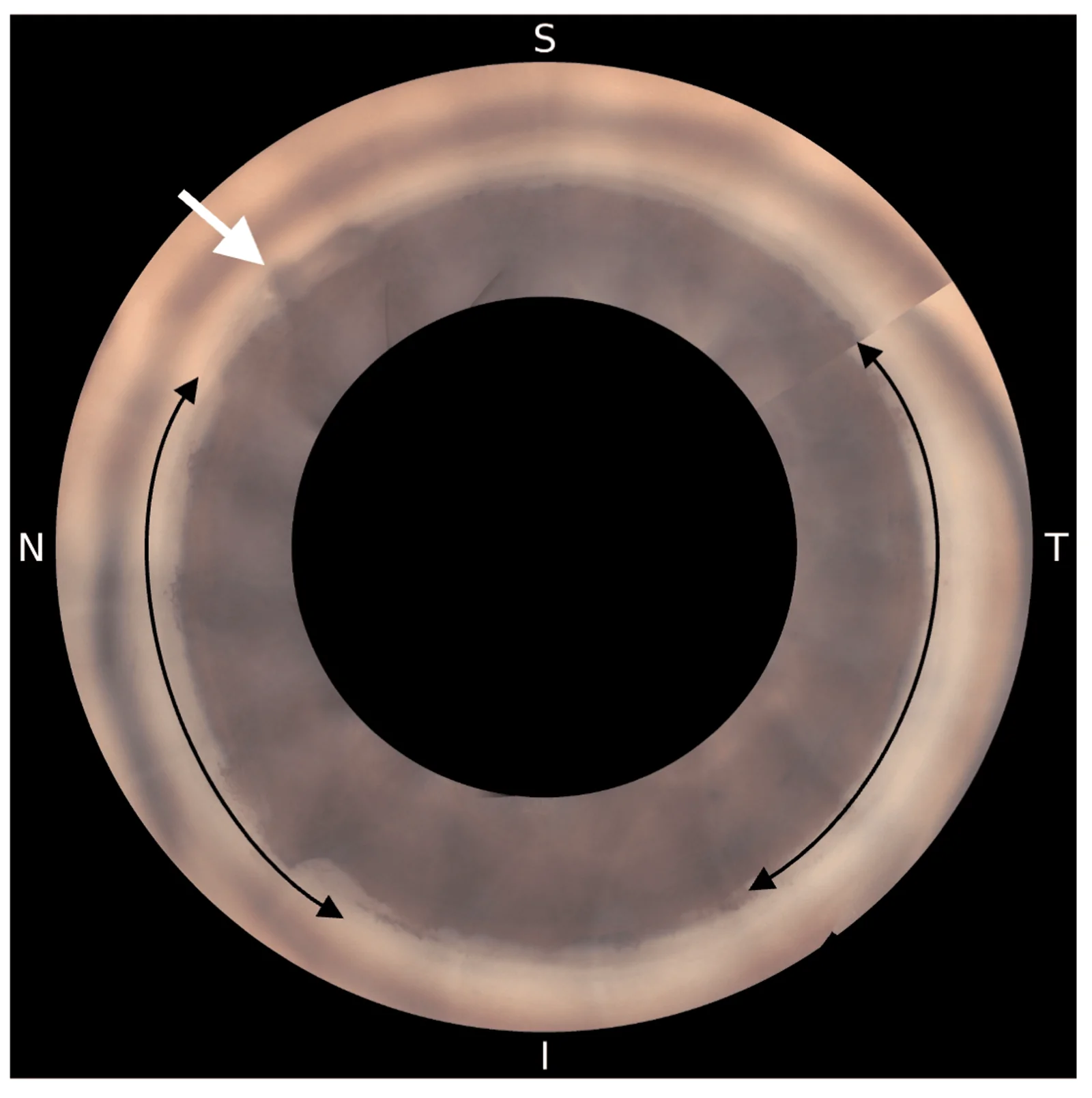
A 360-degree gonioscopic image of the left eye. The previous microhook ab interno trabeculotomy (μLOT) clefts extend from the 2- to 5-o’clock positions temporally and from the 7- to 10-o’clock positions nasally (black curved arrows). The PFM tube entry site at the 11-o’clock position (white arrow) is separate from these clefts. N, nasal; S, superior; T, temporal; I, inferior

At a routine follow-up visit 10 months after AGV implantation, corresponding to two years and three months after PFM implantation, slit-lamp examination incidentally revealed a small number of red blood cells floating in the anterior chamber and a well-formed filtering bleb. The PFM tube was located at the 11-o’clock position (Figure 1A). BCVA OS was 0.2, and IOP OS was 10 mmHg while receiving fixed-combination carteolol/latanoprost once daily and fixed-combination brinzolamide/brimonidine twice daily. Optical coherence tomography (OCT; RS-3000 Advance 2, Nidek, Gamagori, Japan) revealed mild macular edema with subretinal fluid OS. The MD on Humphrey 10-2 visual field testing was −27.28 dB. Anterior segment OCT (AS-OCT; CASIA2, Tomey Corp., Nagoya, Japan) showed that the PFM tube position appeared largely unchanged from its position immediately after implantation (Figure 1B). Gonioscopy revealed a wide-open anterior chamber angle with trace pigmentation and no iris or angle neovascularization. The PFM tube was positioned between the pigmented and non-pigmented TM, and reflux bleeding was observed on both sides of the tube entry site (Figure 1C, Video 1). Examination with the GS-1 360-degree gonioscopy system (Nidek, Gamagori, Japan) showed the previous μLOT clefts, created four years before PFM implantation, extending from the 2- to 5-o’clock positions temporally and from the 7- to 10-o’clock positions nasally. These clefts did not overlap with the PFM tube entry site (Figure 2). The mild hyphema was managed with observation alone. Because the reduction in BCVA was considered more likely attributable to macular edema than to hyphema, a sub-Tenon’s injection of 20 mg triamcinolone acetonide was administered OS.

**Video 1:** Gonioscopic observation of reflux bleeding adjacent to the PFM tube entry site in the left eye.

Three months later, corresponding to two years and six months after PFM implantation, BCVA OS was 0.3, and IOP OS was 13 mmHg. The anterior chamber bleeding had resolved, and the subretinal fluid had decreased. At the next follow-up visit, two years and nine months after PFM implantation, the macular edema had improved, and BCVA OS had further improved to 0.6. IOP OS was 9 mmHg while receiving fixed-combination carteolol/latanoprost once daily and fixed-combination brinzolamide/brimonidine twice daily. Blood deposits were observed on the posterior corneal surface at this visit, suggesting possible recurrent anterior chamber bleeding.

## Discussion

In this case, delayed-onset hyphema was observed more than two years after PFM implantation. The PFM tube position appeared unchanged on AS-OCT, and the previous μLOT clefts did not overlap with the PFM tube entry site. In addition, gonioscopy demonstrated reflux bleeding from both sides of the tube entry site. These findings localized the bleeding to the area adjacent to the PFM tube entry site, with no apparent tube displacement or bleeding from the previous μLOT clefts. Gonioscopy directly identified the site and direction of the reflux bleeding, whereas its origin from SC was determined based on the anatomical location of the tube entry site and the absence of bleeding from the previous μLOT clefts or other angle structures. No rubeosis iridis, abnormal angle scarring, or signs of intraocular inflammation were observed.

Delayed-onset hyphema after μLOT has been reported months to years after surgery, and blood reflux from SC or collector channels has been proposed as a possible mechanism [6]. Because the PFM tube was positioned between the pigmented and non-pigmented TM in the present case, a similar reflux mechanism may have contributed to the delayed-onset hyphema. The observation of bleeding from both sides of the tube entry site more than two years after implantation suggests that small spaces around the tube may have remained patent, allowing blood to enter the anterior chamber. This may reflect limited local scar formation around the tube. Such an interpretation is consistent with experimental findings for poly(styrene-b-isobutylene-b-styrene) (SIBS), the material used in PFM: a rabbit study demonstrated noncontinuous collagen deposition and no detectable macrophages or myofibroblasts around SIBS tubes [7]. However, neither the persistence of these spaces nor the extent of local fibrosis was directly confirmed in the present case. At two years and nine months after PFM implantation, blood deposits were observed on the posterior corneal surface after the initial anterior chamber bleeding had resolved. This finding suggested possible recurrent bleeding through a similar pathway, although gonioscopy was not performed at that visit and recurrence from the same site could not be confirmed.

The cause of visual decline in this case also warrants consideration. Previous reports have described transient IOP spikes associated with hyphema after trabeculotomy [8,9]. In the present case, however, the hyphema was mild, and IOP was 10 mmHg while receiving multiple topical medications when the anterior chamber bleeding was detected. No associated IOP spike was documented. At two years and three months after PFM implantation, BCVA had decreased to 0.2 OS in the presence of both mild hyphema and macular edema. Visual acuity subsequently improved as the macular edema improved following a sub-Tenon’s injection of 20 mg triamcinolone acetonide. This clinical course suggests that macular edema contributed to the transient decrease in visual acuity, although the relative contributions of macular edema and hyphema could not be determined.

The possibility that intraocular blood reflux affected long-term bleb function cannot be excluded. However, the elevated IOP requiring needling and subsequent AGV implantation preceded the documented episode of delayed-onset hyphema. Therefore, the observed bleeding cannot explain the earlier deterioration in IOP control, and any relationship between reflux bleeding and long-term bleb function remains speculative. Further studies are needed to determine whether delayed reflux bleeding after PFM implantation affects long-term surgical outcomes.

This report has several limitations. First, it describes a single case, which limits the generalizability of the findings. Second, although gonioscopy clearly localized the reflux bleeding to both sides of the PFM tube entry site and no other bleeding source was identified, the anatomical communication between SC and the space surrounding the tube was not directly visualized. Third, the absence of gonioscopic examination at the time of suspected recurrence precluded confirmation of recurrent bleeding from the same site. Finally, the history of μLOT and subsequent AGV implantation with pars plana vitrectomy complicates the attribution of delayed-onset hyphema solely to PFM implantation. Despite these limitations, this case documents delayed-onset hyphema occurring more than two years after PFM implantation and shows that careful observation, particularly gonioscopic examination, can help identify the source of bleeding.

## Conclusions

Delayed-onset hyphema was observed more than two years after PFM implantation in this case. Gonioscopy directly demonstrated reflux bleeding from both sides of the PFM tube entry site, supporting reflux from SC through the area surrounding the tube as the mechanism most consistent with the observed bleeding. Although the anatomical communication between SC and the space surrounding the tube was not directly visualized, no other bleeding source was identified. When evaluating similar cases, other potential causes, including iris neovascularization, intraocular inflammation, anticoagulant therapy, angle abnormalities, bleb-related factors, microtrauma, and blood reflux through previously operated sites, may need to be considered. Because management may vary according to the suspected cause and severity, possible approaches range from observation to treatment of an underlying condition or anterior chamber washout. In the present case, the mild hyphema resolved without specific treatment, although possible recurrence was observed. Careful observation, particularly gonioscopic examination, was useful in identifying the bleeding site in this case.
